# Protective effect of *Danggui* (Radix Angelicae Sinensis) on angiotensin II-induced apoptosis in H9c2 cardiomyoblast cells

**DOI:** 10.1186/1472-6882-14-358

**Published:** 2014-09-25

**Authors:** Chih-Yang Huang, Wei-Wen Kuo, Chia-Hua Kuo, Fuu-Jen Tsai, Peng-Yu Liu, Dennis Jine-Yuan Hsieh

**Affiliations:** Graduate Institute of Chinese Medical Science, China Medical University, Taichung, Taiwan; Graduate Institute of Basic Medical Science, China Medical University, Taichung, Taiwan; Department of Health and Nutrition Biotechnology, Asia University, Taichung, Taiwan; Department of Biological Science and Technology, China Medical University, Taichung, Taiwan; Department of Sports Sciences, University of Taipei, Taipei, Taiwan; School of Medical Laboratory and Biotechnology, Chung Shan Medical University, No.110, Sec.1, Jianguo N. Rd., Taichung, 40201 Taiwan; Clinical Laboratory, Chung Shan Medical University Hospital, Taichung, Taiwan

**Keywords:** Traditional Chinese Medicine (TCM), *Danggui*, Radix Angelicae Sinensis, Angiotensin II, Cardiomyoblast, Apoptosis

## Abstract

**Background:**

*Danggui* (Radix Angelicae Sinensis) is an herb often used in Traditional Chinese medicine. It is used to promote blood flow and has been used in the treatment of myocardial ischemia-reperfusion injury in animal models. Angiotensin II (Ang II) has been shown to play important roles in mediating cardiovascular diseases, and may cause cardiac hypertrophy and apoptosis. This study aimed to investigate whether *Danggui* has protective effects on Ang II-induced apoptosis in H9c2 cardiomyoblast cells and study the mechanisms involved.

**Methods:**

We evaluated the effect of *Danggui* on Ang II-induced apoptosis in an *in vitro* model. H9c2 cardiomyoblast cells were cultured in serum-free medium for 4 hr, then treated with *Danggui* (50, 100 μg/ml) 1 hr pre- or post-Ang II treatment. After a further 23 hr of culture, cells were harvested for analyses with assays for apoptosis markers and cell signaling pathways.

**Results:**

Our results showed that Ang II induced upregulation of pro-apoptotic Bad, instability of the mitochondria membrane potential, cytochrome *c* release, caspase-9 and caspase-3 activation and cardiomyocyte apoptosis. Pre- or post-treatment with *Danggui* reversed all of the above Ang II-induced apoptotic effects in H9c2 cells. Furthermore, the JNK (SP600125) inhibitor completely blocked *Danggui* inhibition of caspase-3 activation in Ang II-treated H9c2 cells.

**Conclusions:**

Our results showed that *Danggui* either pre-treatment or post-treatment highly attenuated the Ang II-induced apoptosis in cardiomyoblast cells. The findings demonstrated that the anti-apoptosis effect of *Danggui* is mediated by JNK and PI3k inhibitors.

**Electronic supplementary material:**

The online version of this article (doi:10.1186/1472-6882-14-358) contains supplementary material, which is available to authorized users.

## Background

Cardiovascular disease is a major public health threat for both men and women in many countries. It remains the leading cause of death and disability worldwide. Many factors, such as metabolic syndrome, diabetes mellitus and hypertension, contribute to cardiovascular disease. Several clinical studies have reported that metabolic alterations in diabetes mellitus are associated with modification of growth hormone (GH) and insulin-like growth factor-I (IGF-I) synthesis [1] and may play a role in the pathogenesis of heart failure [2]. In addition, IGF-II and IGF-II receptor (IGF-IIR) have been shown to be associated with the development of cardiac hypertrophy [3–6].

Patients with diabetes often show reduced circulating levels of IGF-I and thus develop IGF-I resistance [7, 8]. This situation may induce apoptosis of myocardial cells and increase the risk of a heart attack. Studies in our laboratory have shown that the mechanism of apoptosis is synergistically mediated by angiotensin II- (Ang II) and the IGF-IR resistance-activated IGF-IIR signaling pathway [5, 9]. This suggests that IGF-IIR and its downstream signaling are important in myocardial apoptosis, and suppression of IGF-IIR signaling pathways can protect myocardial cells from apoptosis.

When the heart receives external stimulation, myocardial cells secrete Ang II [10, 11], which may lead to hypertrophy and apoptosis of the cells [12, 13]. Previously, by using an *in vitro* myocardial cell culture and an animal hypertension model (by abdominal aorta ligation resulting in an elevation in Ang II), we found that Ang II induces IGF-II and IGF-IIR gene expression that is mediated by JNK and ERK activation, and sequentially, IGF-IIR induces Gαq, PKC-α/CaMKIIc and calcineurin activation [9]. The IGF-II-stimulated myocardial cells tend towards pathological hypertrophy, as well as the activation of calcineurin/Bad, resulting in mitochondrial-dependent apoptosis [5]. Many natural products such as herbs have been used in the treatment of heart disease. In this study, we focused on the IGF-IIR-associated pathway, and investigated whether inhibition of Ang II-induced cell damage can reduce cardiac apoptosis.

Radix Angelicae Sinensis (*Danggui*), the dried root of *Angelica Sinensis*, is an herb often used in Chinese food and Traditional Chinese medicine (TCM). Based on early Chinese medical literature, *Danggui* can promote blood flow and has been used to treat disorders with blood deficiency (for review see [14]). It is often used in the treatment of gynecological conditions that possess a blood deficiency pattern, such as dysmenorrhea and an irregular menstrual cycle. *Danggui* has also been reported to have an immunostimulatory effect in a mitogen-stimulated lymphocyte proliferation assay in a mouse model *in vitro* [15]. In addition, it has been shown to increase nitric oxide (NO) formation and vascular relaxation in the rat aorta [16]. It is often tied in with the *qi* drugs (medicinals for treating disordered flow of *qi*, including stagnant flow and counterflow), used to treat the symptoms of blood stasis.

More than 70 compounds, including alkyl phthalides, benzenoids, butylphthalide, coumarins, flavones, organic acids, polysaccharides and trepenes, have been isolated from roots of Angelica sinensis. Among the identified main compounds, Z-ligustilide, phthalides and ferulic acid are known to be its major essential active components [17, 18]. Modern research has also used *Danggui* extract for the treatment of myocardial ischemia-reperfusion injury in rats and found that it has a significant protective effect [19]. Meanwhile, the molecular pathway of *Danggui* in myocardial protection is still unclear and further studies are necessary to clarify the mechanisms underlying the effects of *Danggui*. Therefore, we speculated that *Danggui* may also have a protective effect against Ang II-induced myocardial apoptosis, and aimed to study the associated molecular signaling pathway.

## Methods

### *Danggui*extract

*Danggui* extract powder was provided by Ko Da Pharmaceutical (Taoyuan, Taiwan). After the powder was dissolved in double-distilled water completely, the solution was centrifuged and sterilized by filtering through a 0.2-μm syringe filter and stored at −80°C.

### Cell culture and treatments

H9c2 cardiomyoblasts were obtained from the American Type Culture Collection (ATCC) and cultured in Dulbecco’s modified essential medium (DMEM) supplemented with 10% fetal bovine serum, 2 mM glutamine, 100 units/ml penicillin, 100 μg/ml streptomycin, and 1 mM pyruvate in humidified air (5% CO_2_) at 37°C. To investigate the protective mechanisms of *Danggui* extract against cardiomyocyte apoptosis, H9c2 cardiomyoblast cells were cultured in serum-free medium for 4 hr, followed by treatment with *Danggui* extract (0, 50, 100, 250, 500 and 1000 μg/ml) 1 hr before or after Ang II treatment, respectively (Additional file 1: Figure S1). Cell lysate was collected for analysis 24 hr after Ang II treatment.

### MTT (3-(4,5-Dimethylthiazol-2-yl)-2,5-diphenyltetrazolium bromide) assay

In order to test the cytotoxicity of *Danggui* extract on H9c2 cardiomyoblast cells, H9c2 cells were treated with different concentrations of *Danggui* extract (0, 50, 100, 250, 500, 1000 μg/ml) and the survival rate of H9c2 cells were measured by MTT assay (M5655, Sigma, St. Louis, MO, USA) according to the manufacturer’s instructions.

### DAPI staining and TUNEL assay

After treatment, H9c2 cells were fixed with 4% paraformaldehyde solution for 30 min at room temperature, and permeabilized with 0.1% Triton X-100 for 2 min. Following washing with PBS, samples were first incubated with Terminal Deoxynucleotide Transferase-mediated dUTP Nick End Labeling (TUNEL) (11684817910, Roche, Mannheim, Germany) reagent containing terminal deoxynucleotidyl transferase and fluorescent isothiocyanate-dUTP. The cells were also counterstained with 1 μg/ml DAPI for 30 min. Samples were analyzed under a fluorescence microscope. All morphometric measurements were performed using at least three independent individual samples in a blinded manner.

### JC-1 staining

JC-1 (5, 5′, 6, 6′-tetrachloro-1, 1′, 3, 3′-tetraethylbenzimidazol-carbocyanine iodide) is a lipophilic fluorescent cation that is incorporated into the mitochondrial membrane, where it can form aggregates due to the state of the physiological membrane potential of the mitochondria. This aggregation changes the fluorescence properties of JC-1, leading to a shift from green to orange fluorescence. Intact living cells stained with JC-1 therefore exhibit a pronounced orange fluorescence of mitochondria, which can be detected by confocal microscopy. Apoptosis results in breakdown of the mitochondrial membrane potential and a subsequent decrease of the orange fluorescence (and a slight increase of the green fluorescence). By this means, apoptotic cells can be easily distinguished from non-apoptotic cells. In brief, after treatment, cells were washed with PBS and incubated with medium containing JC-1 staining reagent at 37°C for 20 min followed by washing with PBS. The mitochondrial membrane potential was detected by confocal microscopy.

### Western blot analysis

Cell lysates were separated by 8–12% gradient SDS-PAGE and transferred onto nitrocellulose membrane. The membrane was blocked in 5% milk for 1 hr, and blotted with primary antibody at 4°C overnight. After incubation with secondary antibody for 2 hr at room temperature, the protein bands were detected by enhanced chemiluminescence (ECL, Santa Cruz Biotechnology, Santa Cruz, CA, USA). Densitometric analysis of immunoblots was performed using the LAS-3000 imaging system (Fuji Photo Film, Tokyo, Japan). PI3k antibody (610064) was obtained from BD Biosciences (San Jose, CA, USA), p-Akt (44–622) and p-ERK1/2 (44–680) antibodies were obtained from Biosource Int. (Camarillo, CA, USA), p-Bad antibody (9296) was obtained from Cell Signaling Technology (Beverly, MA, USA) and IGF-II antibody (ab63984) was obtained from Abcam Incorporated (Cambridge, MA, USA). Other monoclonal antibodies were purchased from Santa Cruz Biotechnology (Santa Cruz).

### Inhibitors

H9c2 cells were treated with several inhibitors, including U0126 (MEK1 and MEK2 inhibitor, #V1121, Promega, Madison, WI, USA) and SP600125 (JNK inhibitor) (#1496, TOCRIS /Ellisville, MO, USA).

### Statistical analysis

Statistical differences were assessed by one way-ANOVA using the Duncan test for comparison between groups. *P* < 0.05 was considered statistically significant. Data were expressed as the mean ± SEM.

## Results

### Effects on the cell viability of cells treated with Ang II

**Figure 1:**
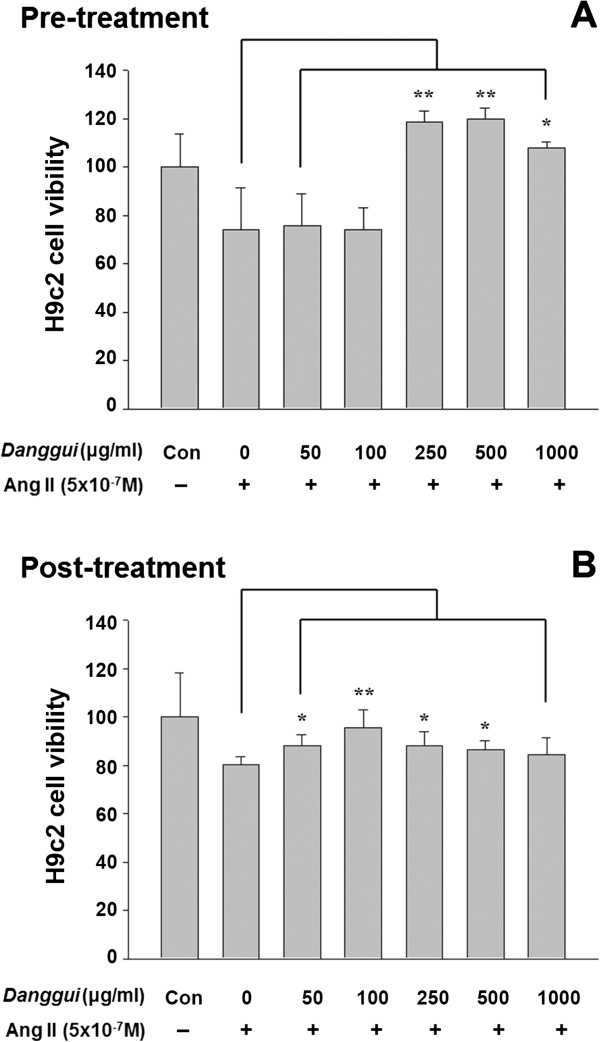
***Danggui*** **extract restores the Ang II-suppressed H9c2 cardiomyoblast cell viability.** The cell viability of Ang II-treated H9c2 cells pre-treated **(A)** and post-treated **(B)** with *Danggui* extract was measured by MTT assay. The results are expressed as the mean ± S.E.M. of four independent experiments. *P < 0.05, **P < 0.01 represent significant differences between cells treated with Ang II alone and cells treated with Ang II and *Danggui* extract.

Pre-treatment with *Danggui* extract at a concentration of 250 μg/ml or higher showed an attenuating effect on Ang II-induced cell death (Figure 1A), while post-treatment with *Danggui* extract also reduced the cell death, but had a lesser effect than pre-treatment (Figure 1B). We also analyzed the cytotoxicity of *Danggui* extract on H9c2 cells, and found that *Danggui* extract has no cytotoxic effect on H9c2 cells at concentrations up to 500 μg/ml, but may cause obvious cell death at concentrations higher than 500 μg/ml after 24 hr of incubation (data not shown). Based on this result, we used *Danggui* extract in the range of 50–500 μg/ml in the following experiments.

### Inhibition of Ang II-induced H9c2 cardiomyoblast apoptosis and DNA fragmentation

**Figure 2:**
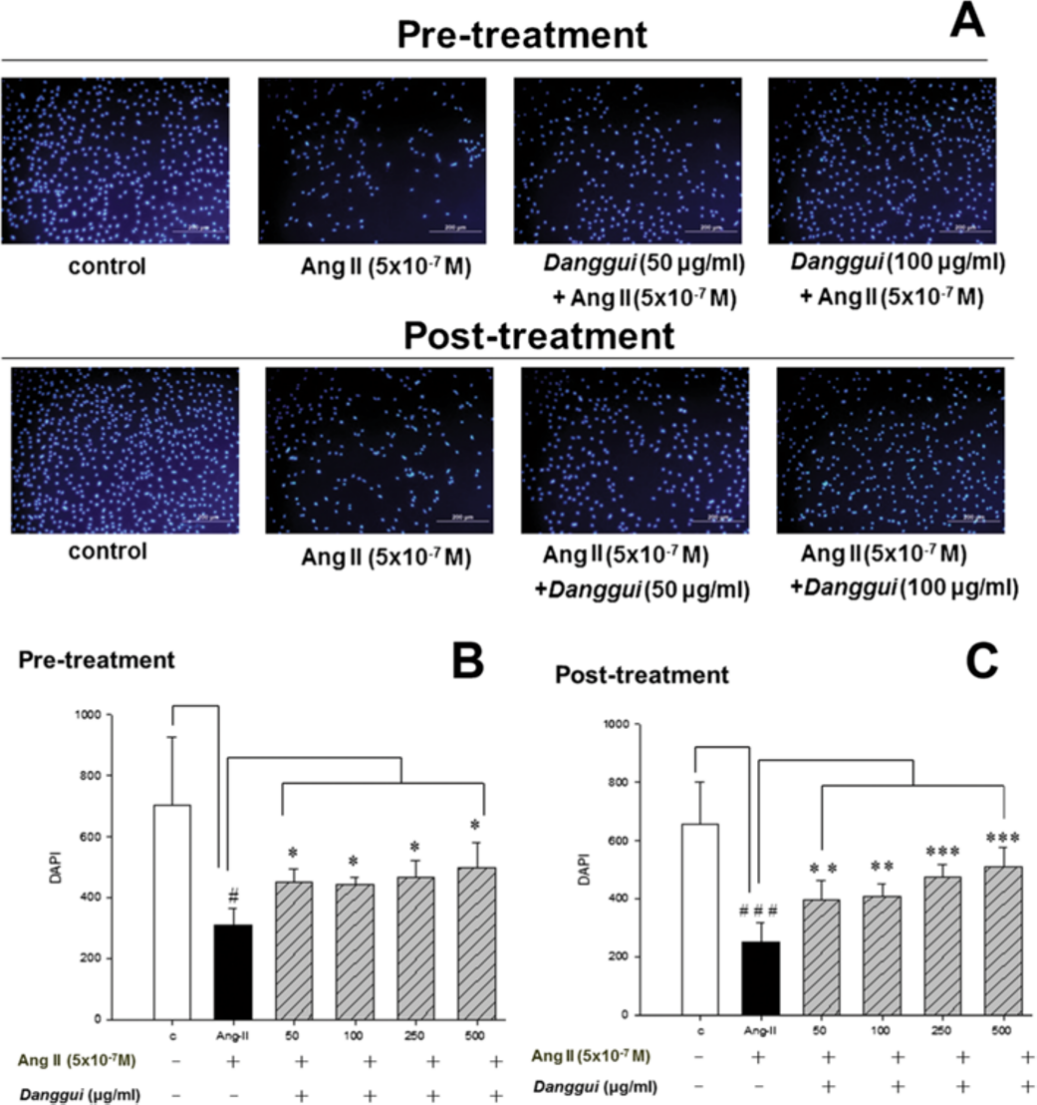
***Danggui*** **extract inhibits the Ang II-induced H9c2 cardiomyoblast cells apoptosis. (A)** Influence of pre-treatment or post-treatment with *Danggui* extract on Ang II-induced H9c2 cardiomyoblast apoptosis. The blue spot indicates DAPI staining representing the cell number. Percentage of apoptotic cells following pre-treatment **(B)** or post-treatment **(C)** with *Danggui* extract in the control and Ang II-treated H9c2 cells. The results are expressed as the mean ± S.E.M. of four independent experiments. ^#^ *P* < 0.05, ^###^ *P* < 0.001 between the controls and cells treated with Ang II alone. *P < 0.05, **P < 0.01, ***P < 0.001 between cells treated with Ang II alone and cells treated with Ang II and *Danggui* extract.

The results of DAPI staining showed that Ang II treatment induced apoptosis in H9c2 cells, while pre-treatment or post-treatment with *Danggui* extract (50 to 500 μg/ml) resulted in a significant decrease of Ang II-induced apoptosis (Figure 2). This result suggested that *Danggui* extract has an inhibition effect against Ang II- induced apoptosis in H9c2 cardiomyoblast cells.

**Figure 3:**
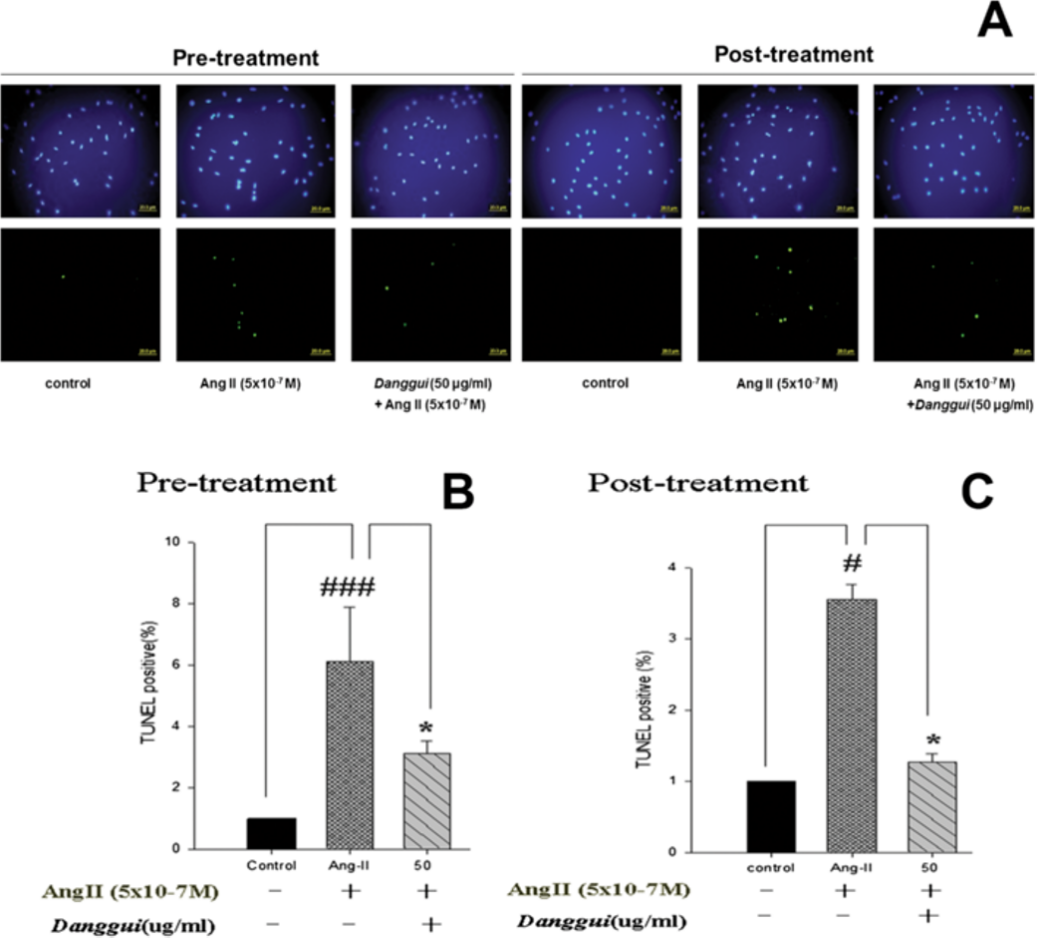
***Danggui*** **extract inhibits Ang II-induced DNA fragmentation in H9c2 cardiomyoblast cells. (A)** Effect of pre-treatment or post-treatment with *Danggui* extract on Ang II-induced H9c2 cardiomyoblast apoptosis. The blue spot indicates DAPI counterstaining representing the cell number, and the green spot represents the apoptotic cells stained by TUNEL assay. Percentage of apoptotic cells following pre-treatment **(B)** or post-treatment **(C)** with *Danggui* extract in Ang II-induced H9c2 cells. The results are expressed as the mean ± S.E.M. of four independent experiments. ^#^ *P* < 0.05, ^###^ *P* <0.001 in relation to the control. *P < 0.05 in relation to the H9c2 cells treated with Ang II alone.

We then used a TUNEL assay to analyze the apoptosis level in H9c2 cells with a DAPI counterstain to calculate the total cell number. The result showed that the number of apoptotic bodies was increased with Ang II treatment, and was significantly decreased with pre-treatment or post-treatment with *Danggui* extract (at as low as 50 μg/ml) (Figure 3).

### Inhibition of Ang II-induced caspase-9 and caspase-3 activation

**Figure 4:**
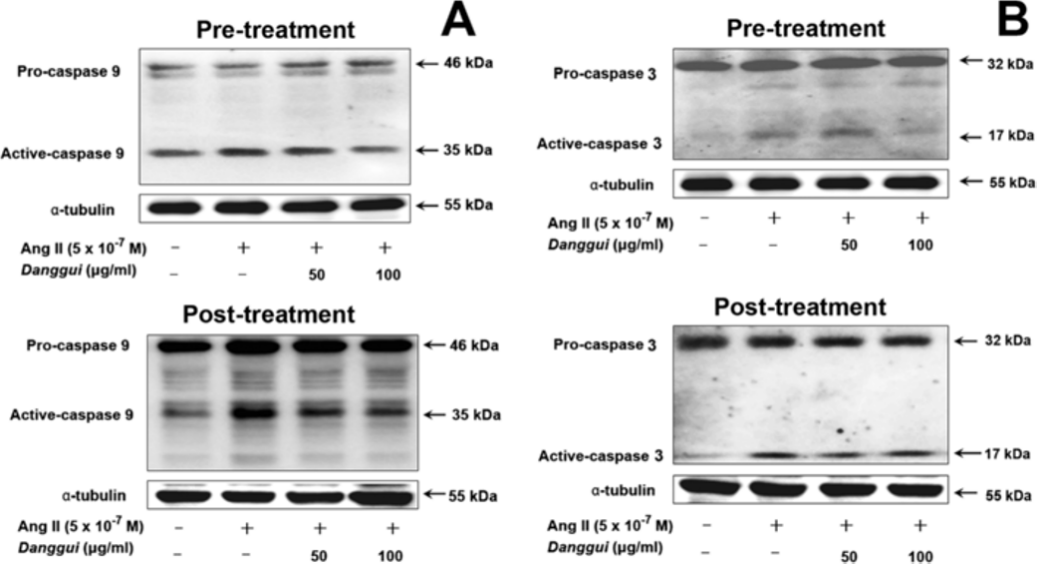
***Danggui*** **extract inhibits Ang II-induced caspase 9 (A) and caspase 3 (B) activation in H9c2 cardiomyoblast cells.** H9c2 cells were treated with Ang II (5×10^−7^ M) for 1 hr, and then treated with *Danggui* extract (50, 100 μg/ml) either 1 hr before (pre-treatment) or 1 hr after (post-treatment) Ang II treatment, followed by another 23 hr of culture. Cells were harvested and extracted for Western blotting analysis. α-tubulin was employed as the internal control.

To investigate the involvement of caspases in Ang II-induced cell death, we performed Western blotting analysis of caspase-9 and caspase-3. As shown in Figure 4, the protein levels of active caspase-9 and caspase-3 were increased with Ang II treatment, and were significantly decreased following both pre-treatment and post-treatment with *Danggui* extract in H9c2 cells. This suggests that *Danggui* extract can downregulate the mitochondrial death pathway to inhibit the apoptosis induced by Ang II. All the Western data are quantified and the results are showed in Supplementary data (Additional file 1: Figure S2).

### Rescue effect on the Ang II-induced instability of the mitochondria membrane potential and cytochrome *c*release

**Figure 5:**
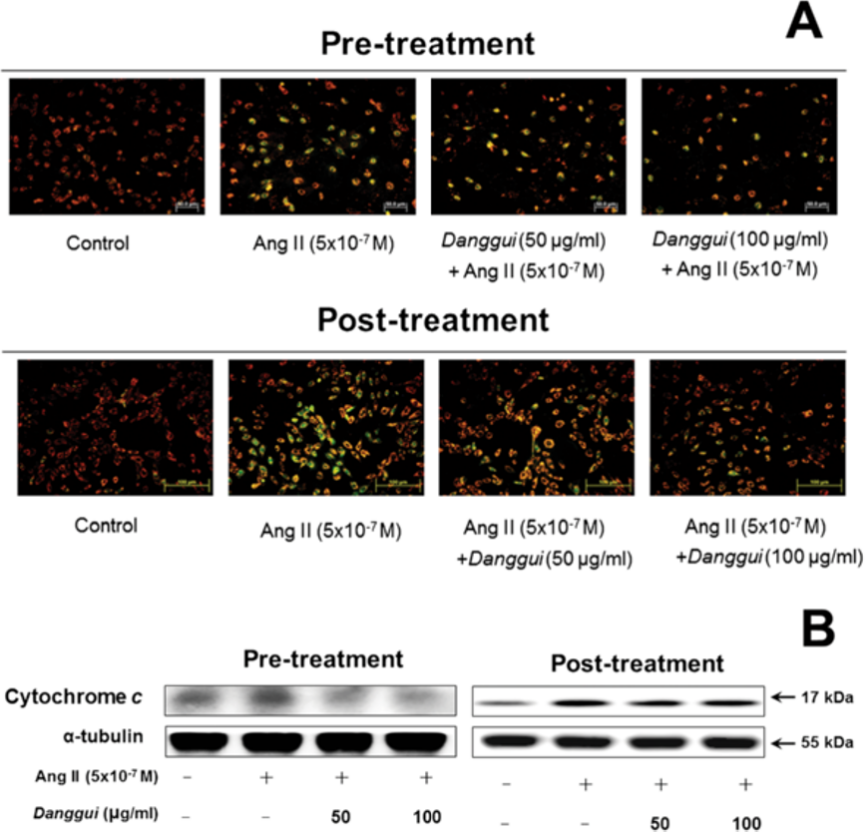
***Danggui*** **extract reverses the Ang II-induced instability of the mitochondria membrane potential and cytochrome** ***c*** **expression. (A)** Ang II induced instability of the mitochondria membrane potential, as detected by Fluorescein isothiocyanate- (FITC-) JC-1 staining in H9c2 cells. The red image represents that the stable state of *Danggui* extract could stabilize the mitochondria membrane potential and the green image shows the cells in an unstable state. **(B)** H9c2 cells were treated with Ang II (5x10^−7^ M) for 1 hr, and then treated with *Danggui* extract (50, 100 μg/ml) either 1 hr before (pre-treatment) or 1 hr after (post-treatment) Ang II treatment, followed by another 23 hr of culture. The protein expression of cytochrome *c* was assessed by Western blotting. α-tubulin was employed as the internal control.

In order to validate the involvement of the mitochondrial death pathway in H9c2 cells after Ang II treatment, the mitochondrial membrane potential was assessed by JC-1 staining. The result demonstrated that Ang II caused the mitochondria membrane potential to become more unstable (Figure 5A) and led to cytochrome *c* release (Figure 5B). However, when Ang II-treated H9c2 cells were pre-treated or post-treated with *Danggui* extract, the mitochondria membrane potential stabilized and the release of the mitochondria membrane protein cytochrome *c* decreased (Figure 5).

### Signaling mechanism of the anti-apoptosis effect in H9c2 cardiomyoblast cells

**Figure 6:**
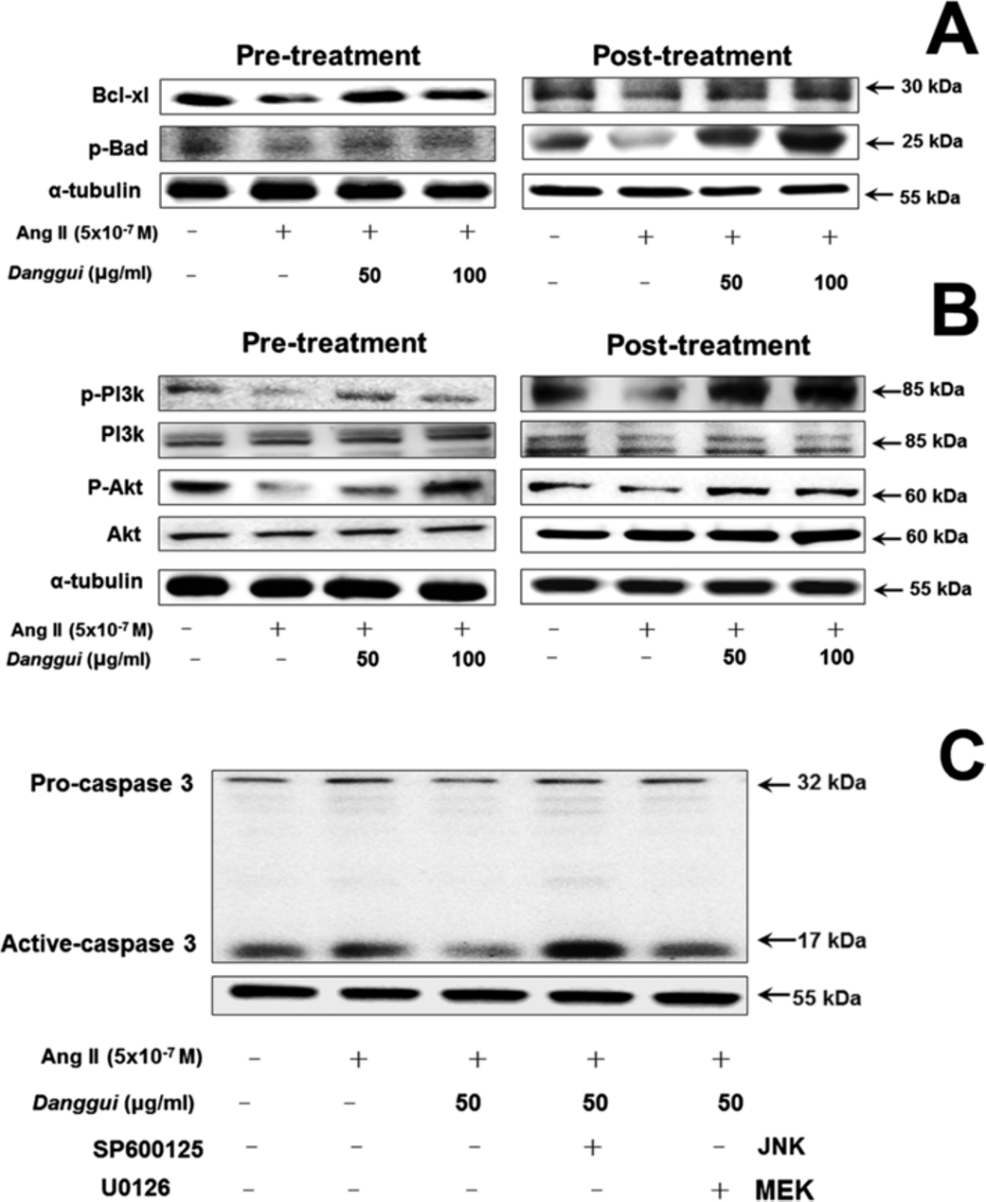
***Danggui*** **extract reversed the Ang II-reduced cardiac survival pathway in H9c2 cardiomyoblast cells.** H9c2 cells were treated with Ang II (5×10^−7^ M) for 1 hr, and then treated with *Danggui* extract (50, 100 μg/ml) either 1 hr before (pre-treatment) or 1 hr after (post-treatment) Ang II treatment, followed by another 23 hr of culture. Western blotting analysis of **(A)** Bcl-x_L_ and p-Bad expressions, and **(B)** p-PI3K, PI3K, p-Akt and Akt. **(C)** pro-caspase-3 and caspase-3 expression in H9c2 cells treated with inhibitors U0126 (MEK1 and MEK2 inhibitor) and SP600125 (JNK inhibitor). α-tubulin was used as the internal control.

To understand the mechanism controlling mitochondrial permeability, we analyzed Bcl-2 family proteins, which are key regulators and may be involved in the apoptosis of cardiomyoblast cells. Our results showed that the levels of anti-apoptotic proteins phosphorylated Bad (p-Bad) and Bcl-x_L_ were decreased by Ang II treatment, and pre- or post-treatment with *Danggui* extract rescued the expressions of Bad (p-Bad) and Bcl-x_L_ (Figure 6A). These results indicated the anti-apoptotic potential of *Danggui* extract, which contributes to stabilizing the mitochondria membrane potential through regulating related proteins against apoptosis.

In order to elucidate whether *Danggui* extract inhibits apoptosis by activating the cardiac IGF-I survival pathway, phosphatidylinositol 3-kinase (PI3k) and Akt kinase (Akt) proteins were analyzed by Western blot. It was found that the phosphorylated PI3k (p-PI3k) and phosphorylated Akt (p-Akt) protein levels were greatly decreased by Ang II, but the protein levels could be recovered by both pre-treatment and post-treatment treatment with *Danggui* extract in H9c2 cells (Figure 6B).

The chemical inhibitors JNK (SP600125) and MEK (U0126) were applied to investigate the mediator for *Danggui* extract-attenuated Ang II-stimulated caspase-3 activation. The results showed that the JNK inhibitor (SP600125) totally blocked *Danggui* extract-inhibited caspase-3 activation in Ang II-treated H9c2 cells, but the MEK inhibitor (U0126) did not (Figure 6C).

## Discussion

*Danggui* is a key herb in several herbal formulas that are used to treat several diseases. These *Danggui*-containing formulas, such as *Danggui* Buxue Decoction, have been shown to have an antioxidant effect [20, 21]. *Danggui* has also been shown to promote angiogenesis [22]. *Danggui* is not only commonly used to treat various gynecological conditions, but recent studies have also shown that it can prevent doxorubicin-induced chronic cardiotoxicity and reduce myocardial injury in animal models [23, 24]. A range of active compounds have already been isolated and identified from *Danggui* [17]. Among the identified main compounds, Z-ligustilide, phthalides and ferulic acid are known to be its major essential active components [17, 18].

Ang II has been shown to be a risk factor for cardiovascular diseases, as it has been reported to cause cardiac hypertrophy and apoptosis. Our previous study demonstrated that Ang II may evoke IGF-II and IGF-IIR through the ERK and JNK signaling pathways, and further activates cardiac cell apoptosis via calcineurin-dependent pathways [9]. This might be the key step that causes heart failure. Furthermore, a study performed in our laboratory demonstrated that pathological hypertrophic stimulus, including Ang II, up-regulates the IGF-IIR gene expression in H9c2 cells, and histone acetylation plays a critical role in IGF-IIR up-regulation [25]. We also noticed that the Ang II-induced IGF-IIR gene expression can be reversed by *Danggui* treatment (data not shown). Thus, we speculated that *Danggui* may prevent the Ang II-induced damage in cells.

MTT assay showed that *Danggui* extract has no cytotoxic effect on H9c2 cells at concentrations up to 500 μg/ml. Ang II administration caused a significant decrease in the cell viability (Figure 1), which is in agreement with the results of another study [26]. Ang II has been shown to inhibit the IGF-IR signaling pathway and activate the IGF-IIR signaling pathway in damaged H9c2 cells [9, 27]. The results of the current study showed that *Danggui* extract at a concentration between 100 and 500 μg/ml prevents the H9c2 cell damage caused by Ang II treatment.

It has been shown previously that cell survival is decreased in Ang II-treated H9c2 cells [27], and in this study, we showed that pre-treatment or post-treatment with *Danggui* extract significantly protected H9c2 cells from Ang II-induced apoptosis. Furthermore, the effects of pre- and post-treatment with *Danggui* extract on Ang II-induced H9c2 cells apoptosis were both effective and dose-dependent (Figure 2B). These results suggested that *Danggui* extract has a highly protective effect against Ang II-induced apoptosis. Ang II stimulation induces apoptosis of ventricular myocytes in several animal models [28, 29]. The results of a TUNEL assay supported that apoptotsis induced by Ang II treatment was significantly inhibited by both pre-treatment and post-treatment with *Danggui* extract.

Ang II activates calcium-calmodulin-dependent protein phosphatase calcineurin through Galphaq (Gα-q)/PLC signaling transduction [27, 30]. Ca^2+^/calcineurin also dephosphorylates pro-apoptotic Bcl-2 family protein Bad and induces cytochrome *c* release from mitochondria to the cytosol, which further induces caspase activation and cardiac cell apoptosis [27, 31]. We showed that Ang II stimulation causes apoptosis and induces caspase-9 and caspase-3 activation (Figure 4). When cells were pre-treated with *Danggui* extract, the protein levels of active caspase-9 and caspase-3 were both significantly decreased at a concentration of 100 μg/ml. Post-treatment with *Danggui* extract still resulted in a significant decrease in the active caspase-9 level.

Furthermore, it was found that *Danggui* extract can block Ang II-induced mitochondria membrane potential instability and cytochrome *c* release. In addition, the effect of pre-treatment with *Danggui* extract was more obvious than that of post-treatment. These findings suggest that the protective effect of *Danggui* extract is mediated by stabilization of the mitochondria membrane potential and inhibition of cytochrome *c* release against Ang II-induced caspase-9 and caspase-3 activation and cell apoptosis. This study reports for the first time that *Danggui* extract treatment significantly reduces Ang II-induced myocyte apoptosis as indicated by the reduction in caspase-9 and caspase-3 activity.

An earlier study showed that retinoic acid improved mitochondrial function by inhibiting the mechanical damage and Ang II-induced reduction in the mitochondrial membrane potential, cytochrome *c* release, and by increasing the Bcl-2/Bax ratio [32]. In addition, previous studies have demonstrated that JNK can suppress apoptosis in IL-3-dependent hematopoietic cells via phosphorylation of the pro-apoptotic Bcl-2 family protein Bad [33], and prevents inactivation of the pro-survival Bcl-2 family protein Bcl-x_L_ by Bad [34]. The results of this study revealed that anti-apoptotic proteins p-Bad and Bcl-x_L_ were decreased by Ang II treatment, and the levels of p-Bad and Bcl-x_L_ were significantly increased after pre- or post-treatment with *Danggui* extract (Figure 6A). These findings indicate that the anti-apoptotic potential of *Danggui* extract is mediated by stabilizing the mitochondrial membrane potential through regulating anti-apoptotic proteins against apoptosis.

**Figure 7:**
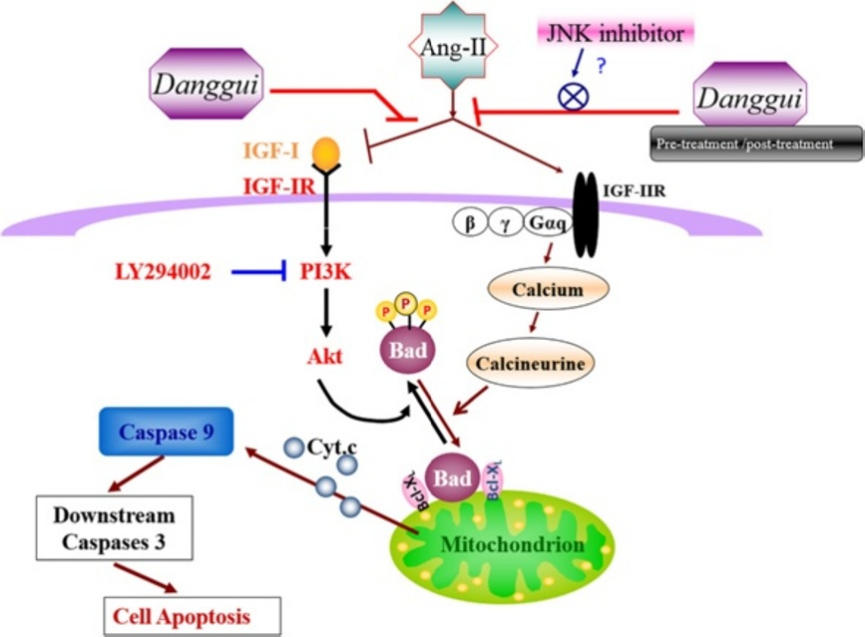
**Schematic diagram.** Possible mechanism of *Danggui*-associated inhibition of the mitochondrial-dependent apoptosis pathway induced by Ang II in H9c2 cardiomyoblast cells.

Kuo and colleagues [27] reported that IGF-I/IGF-IR via intracellular signaling pathways involving tyrosine kinase activity may exert an anti-apoptotic effect via PI3k and Akt-dependent Bad phosphorylation (p-Bad). Therefore, the IGF-I/IGF-IR signaling pathway may play an important role in the Ang II-reduced cardiac survival pathway in H9c2 cells. To clarify whether *Danggui* extract inhibition of apoptosis is mediated by activating this pathway, the levels of p-PI3k and p-Akt were analyzed. Our results demonstrated that the Ang II-induced decreased expressions of these proteins were recovered with both pre-treatment and post-treatment with *Danggui* extract. In addition, angiotensin converting enzyme inhibitor might increase the IGF-I concentration [35] and Ang II might decrease circulating IGF-I in rats [36]. Ang II reduces the IGF-I-stimulated sodium pump activity by attenuating PI3k/Akt signaling in vascular smooth muscle cells [37]. Therefore, we hypothesized that Ang II may block IGF-I expression or enhance IGF-I resistance and inhibit the IGF-IR-mediated PI3k-Akt signaling pathway to induce H9c2 cell apoptosis, which may recover following treatment with *Danggui* extract (Figure 7). We also found that in the cells co-stimulated with Ang II and *Danggui*, SP600125 inhibited JNK activity (decreased the pro-apoptotic response) and activated p-PI3k and p-Akt (increased the anti-apoptotic effect). However, SP600125 alone or treatment with Ang II and *Danggui* both increased the active caspase-3 expression (Additional file 1: Figure S3). The reason for SP600125 (JNK inhibitor) to increase p-Akt activity is still not clear. However, similar finding has been reported in rat granule neurons [38].

As IGF-II and IGF-IIR also play critical roles in Ang II-induced apoptosis in H9c2 cells, it is critical to know which signaling pathways are involved in the *Danggui* extract-mediated protective effect. The JNK inhibitor (SP600125) can block Ang II-induced IGF-IIR expression and apoptosis [9], suggesting that JNK activation mediates Ang II-induced apoptosis. However, the results of the present study also showed that the JNK inhibitor (SP600125) can completely block the inhibition by *Danggui* extract of caspase-3 activation in Ang II-treated H9c2 cells, but the MEK inhibitor (U0126) does not. This indicates that JNK activation mediates the protective effect of *Danggui* extract. Besides, many previous studies have shown that JNK activation mediates anti-apoptotic effects [33, 34, 39]. Taken together, these findings and the results of this study suggest that JNK activation has a dual pro-apoptotic and anti-apoptotic effect on Ang II- and *Danggui* extract-mediated cell viability, respectively, although the underlying molecular mechanism remains to be elucidated. Furthermore, to further investigate the importance of the JNK pathway in *Danggui*-protected Ang II-induced IGF related cardiac apoptosis, we explored the role of the IGF-I/IGF-IR pathway in the *Danggui* extract-mediated protective effect. By Western blotting analysis, we examined the IGF II and IGF IIR expressions and the PI3K/AKT pathway after JNK inhibitor co-treatment with *Danggui* and Ang II treatment in H9c2cells (Additional file 1: Figure S3). Like the JNK inhibitor (SP600125), the PI3k inhibitor (LY294002) completely blocked the inhibition by *Danggui* extract of caspase-3 activation in Ang II-treated H9c2 cells, but the IGF-IR inhibitor (AG1024) did not (Additional file 1: Figure S4). Consequently, according to the inhibitors assay, PI3k activation mediates the protective effect of *Danggui* extract.

## Conclusions

In conclusion, the induction of downregulation of anti-apoptotic p-PI3k, p-Akt and Bcl-x_L_, upregulation of pro-apoptotic Bad, instability of the mitochondria membrane potential, cytochrome *c* release, caspase-9 and caspase-3 activation and cardiomyocyte apoptosis were observed after Ang II treatment. However, both pre- and post-treatment with *Danggui* extract reversed all of the Ang II-induced effects. We have provided herein the first evidence that *Danggui* can inhibit AngII-induced apoptosis, and JNK and PI3k inhibitors can block the anti-apoptosis effect of *Danggui*.

## Electronic supplementary material

Additional file 1: Figure S1: Flow diagram of the study. **Figure S2.** Quantified Western results of Figures 4, 5, 6 (caspase-9, caspase-3, cytochrome c, p-PI3k, p-Akt, Bcl-xl and p-Bad). **Figure S3.** Western blotting analysis of IGF -I, IGF-IIR, Akt and PI3Kk expressions after JNK inhibitor co-treatment with *Danggui* and Ang II in H9c2 cells. **Figure S4.** Western blotting analysis of capase-3 after JNK inhibitor co-treatment with *Danggui* and Ang II in H9c2 cells. (PDF 475 KB)
