# Supplementary figures and images for: Protective effect of Danggui (Radix Angelicae Sinensis) on angiotensin II-induced apoptosis in H9c2 cardiomyoblast cells
Source: BMC Complement Altern Med. 2014 Sep 25;14:358. doi: 10.1186/1472-6882-14-358 (PMC4182826; doi:10.1186/1472-6882-14-358)

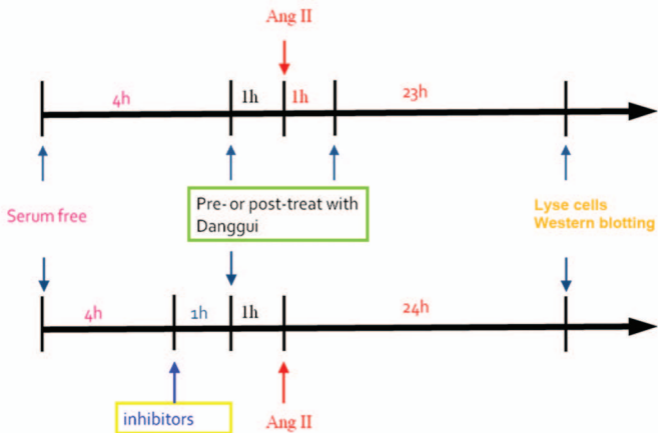

**Figure S1**

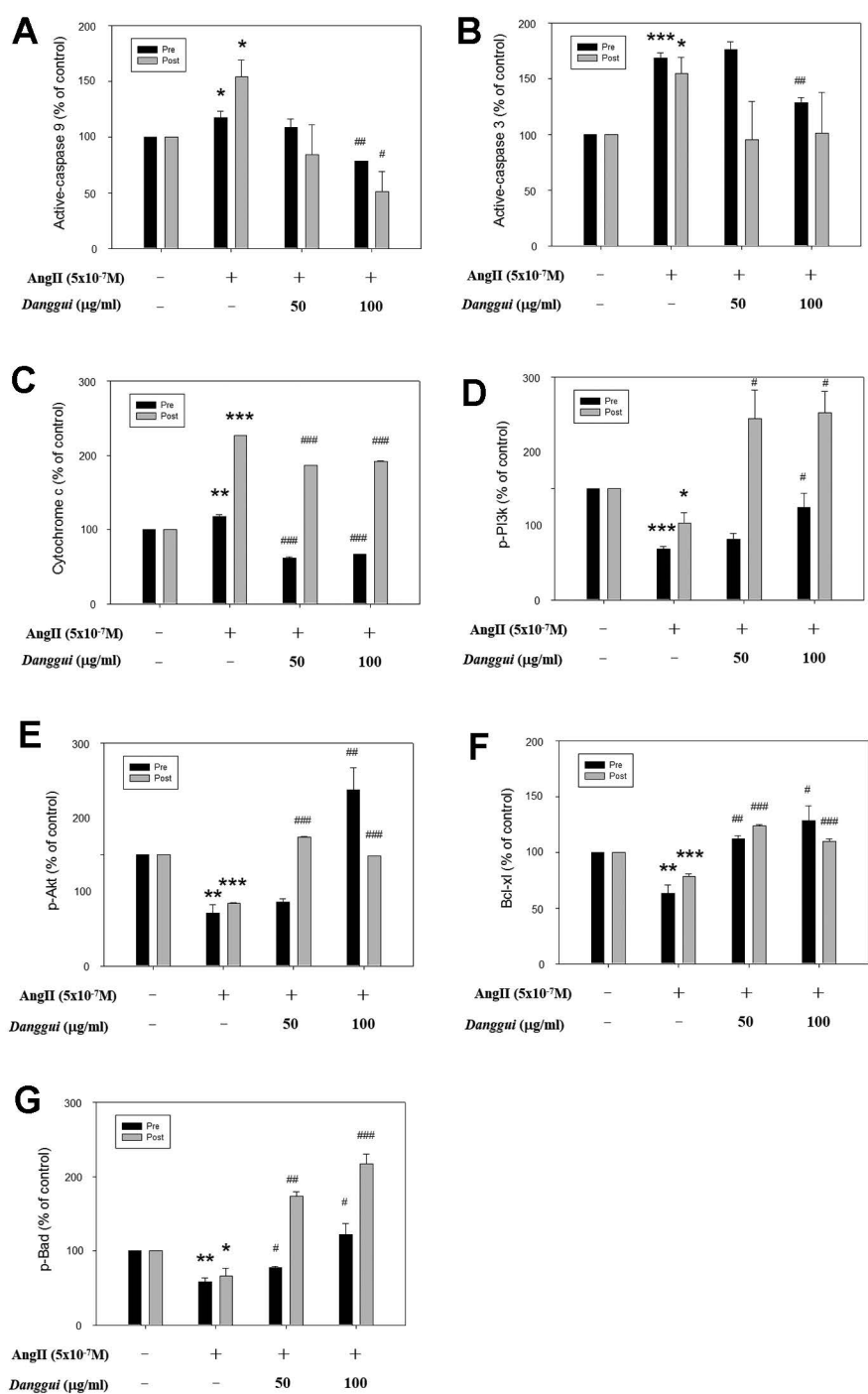

**Figure S2**

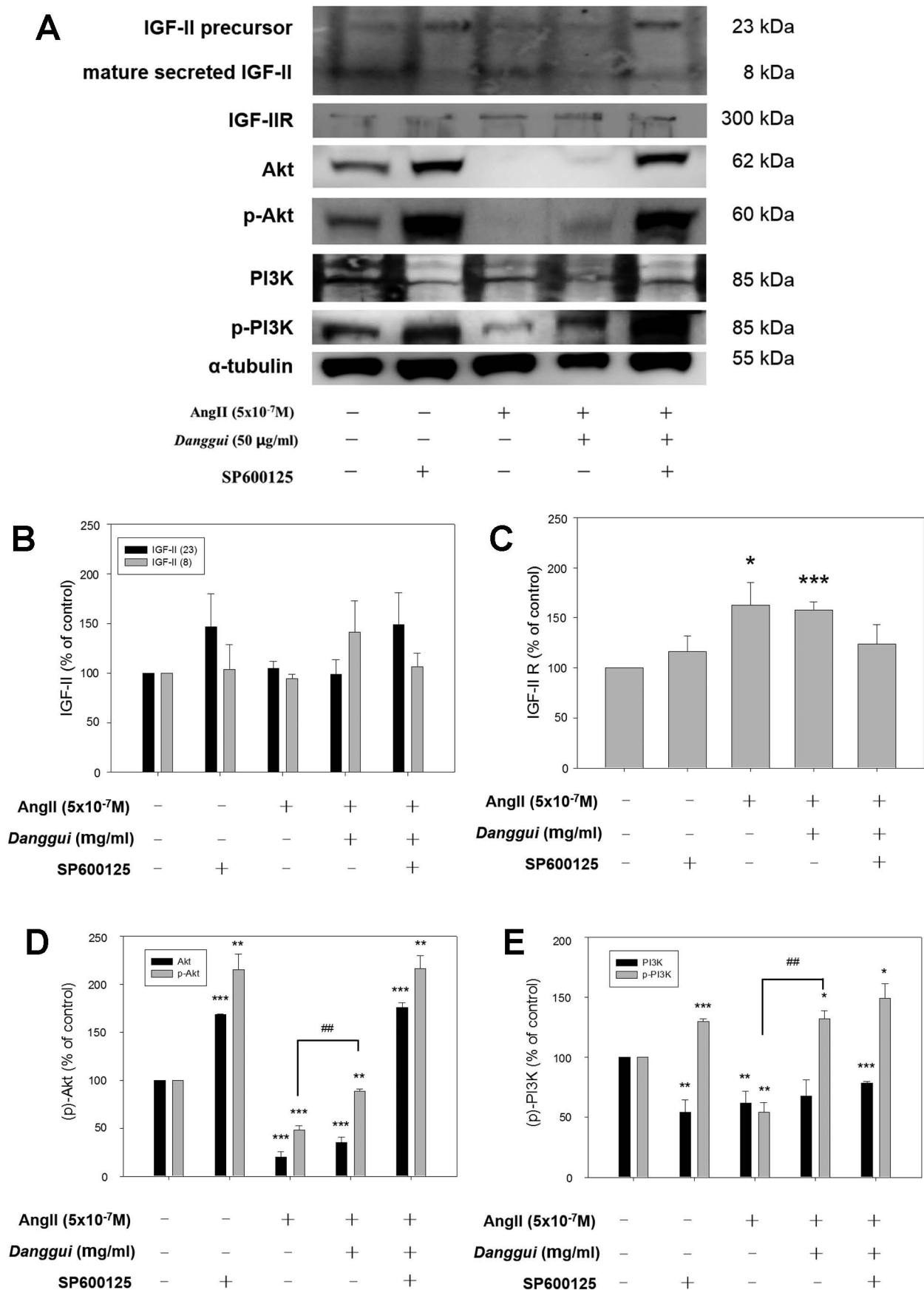

Figure S3

**A**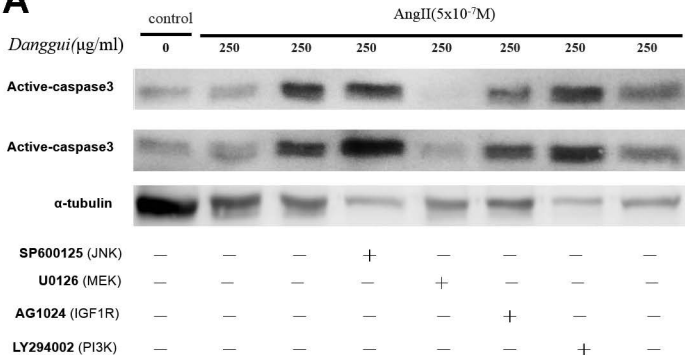**B**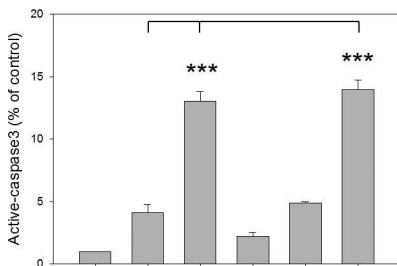

|                                 |   |   |   |   |   |   |
|---------------------------------|---|---|---|---|---|---|
| <i>Danggui</i> (250 $\mu$ g/ml) | — | + | + | + | + | + |
| AngII( $5 \times 10^{-7}$ M)    | — | + | + | + | + | + |
| SP600125 (JNK)                  | — | — | + | — | — | — |
| U0126 (MEK)                     | — | — | — | + | — | — |
| AG1024 (IGF1R)                  | — | — | — | — | + | — |
| LY294002 (PI3K)                 | — | — | — | — | — | + |

**Figure S4**

Supplement: Supplementary file 1 — Additional file 1: Figure S1: Flow diagram of the study. Figure S2. Quantified Western results of Figures 4, 5, 6 (caspase-9, caspase-3, cytochrome c, p-PI3k, p-Akt, Bcl-xl and p-Bad). Figure S3. Western blotting analysis of IGF -I, IGF-IIR, Akt and PI3Kk expressions after JNK inhibitor co-treatment with Danggui and Ang II in H9c2 cells. Figure S4. Western blotting analysis of capase-3 after JNK inhibitor co-treatment with Danggui and Ang II in H9c2 cells. (PDF 475 KB) [file 12906_2014_1929_MOESM1_ESM.pdf]
